# Breast cancer associated germline structural variants harboring small noncoding RNAs impact post-transcriptional gene regulation

**DOI:** 10.1038/s41598-018-25801-1

**Published:** 2018-05-14

**Authors:** Mahalakshmi Kumaran, Preethi Krishnan, Carol E. Cass, Roland Hubaux, Wan Lam, Yutaka Yasui, Sambasivarao Damaraju

**Affiliations:** 1 Department of Laboratory Medicine & Pathology, University of Alberta, Edmonton, Alberta T6G 2R3 Canada; 2 Department of Oncology, University of Alberta, Edmonton, Alberta T6G 2R3 Canada; 3 School of Public Health, University of Alberta, Edmonton, Alberta T6G 2R3 Canada; 4 Cross Cancer Institute, Alberta Health Services, Edmonton, T6G 1Z2 Alberta Canada; 5 Department of Integrative Oncology, British Columbia Cancer Agency, Vancouver, BC Canada

## Abstract

Copy Number Variants (CNVs) are a class of structural variations of DNA. Germline CNVs are known to confer disease susceptibility, but their role in breast cancer warrants further investigations. We hypothesized that breast cancer associated germline CNVs contribute to disease risk through gene dosage or other post-transcriptional regulatory mechanisms, possibly through tissue specific expression of CNV-embedded small-noncoding RNAs (CNV-sncRNAs). Our objectives are to identify breast cancer associated CNVs using a genome wide association study (GWAS), identify sncRNA genes embedded within CNVs, confirm breast tissue (tumor and normal) expression of the sncRNAs, correlate their expression with germline copy status and identify pathways influenced by the genes regulated by sncRNAs. We used an association study design and accessed germline CNV data generated on Affymetrix Human SNP 6.0 array in 686 (in-house data) and 495 (TCGA data) subjects served as discovery and validation cohorts. We identified 1812 breast cancer associated CNVs harboring miRNAs (n = 38), piRNAs (n = 9865), snoRNAs (n = 71) and tRNAs (n = 12) genes. A subset of CNV-sncRNAs expressed in breast tissue, also showed correlation with germline copy status. We identified targets potentially regulated by miRNAs and snoRNAs. In summary, we demonstrate the potential impact of embedded CNV-sncRNAs on expression and regulation of down-stream targets.

## Introduction

Globally, breast cancer (BC) is one of the most common cancers diagnosed among women^1^. It is estimated from twin studies that genetic factors contribute up to 30% of the risk for breast cancer^2^. To date, high, moderate and low penetrance single nucleotide variants associated with breast cancer explained only 50% of the heritable risk and much of the remaining genetic susceptibility (so-called missing heritability) remains unexplored^3,4^. However, majority of these variants are present in the intronic or intergenic regions and therefore precludes delineation of their role in breast cancer pathogenesis. Therefore, there is a need to explore the significance of other forms of genetic variants for their role in breast cancer heritability.

Copy Number Variations (CNVs), are a class of structural variations of DNA (>50 bp in size), which includes amplification or deletion of genomic segments. CNVs can influence phenotype in a variety of ways: through gene dosage (correlation of copy status and ensuing tissue specific gene expression changes), partial deletions in genic regions leading to fusion genes, or complete deletions of genes, and lastly, changes that lead to more complex levels of *cis* or *trans* regulatory functions^5,6^.

Recently, genetic susceptibility has been explained in part by common germline CNVs (>5% in frequency) and rare germline CNVs (1–5% in frequency) for sporadic and familial breast cancers, respectively^6,7^. A common germline CNV deletion affecting *APOBEC3* loci resulted in a fusion protein, *APOBEC3A_B*, which was reported to confer breast cancer susceptibility in diverse populations^6,8,9^. Recently, we demonstrated that germline CNVs overlapping with protein coding genes are associated with breast cancer risk and prognosis. Also the associated CNVs showed gene dosage effects, *i.e*., germline copy status (gain, loss or diploid status) and showed correlation with breast tissue gene expression^7^. Even though previous studies have suggested that a significant proportion of CNVs reside in the intergenic regions which harbor non-coding genes, there were no direct studies to address their relevance to breast cancer. We reasoned that studies of germline CNVs harboring small non-coding RNAs (hereafter referred to as CNV-sncRNAs) such as microRNAs (miRNAs), piwi-interacting RNAs (piRNAs), small nucleolar RNAs (snoRNAs) and transfer RNAs (tRNAs) and their relative levels of expression in breast tissues potentially offers biological insights into the role of CNV-sncRNAs in breast cancer risk.

sncRNAs are less than 200 nucleotides in size and include different classes of RNAs – miRNAs, piRNAs, snoRNAs and tRNAs. While miRNAs and piRNAs are known post-transcriptional regulators of gene expression, snoRNAs and tRNAs are also currently being investigated as potential regulators of gene expression. Although the canonical roles of snoRNAs and tRNAs include RNA modification/splicing and translation, respectively, novel functions of these RNAs are emerging. The nucleotide sequences within these RNAs show sequence homology with mature miRNAs and piRNAs. snoRNAs and tRNAs may undergo nucleolytic processing to unmask cryptic miRNAs and piRNAs. Dysregulation of all four classes of sncRNAs has been observed in various cancer types, including breast cancer, and its clinical significance has been addressed in some detail (miRNAs and piRNAs)^10,11^ or is emerging (snoRNAs and tRNAs)^12,13^.

Germline single nucleotide polymorphisms (SNPs) present in pre-miRNA regions are known to affect their biogenesis and target binding efficiencies of miRNAs, thereby influencing disease predisposition^14–16^. Germline CNVs may also affect disease predisposition by independent mechanisms. For instance, a copy number deletion of a miRNA cluster present on chr22q11.2 locus is a classic example of a germline CNV as a genetic determinant of schizophrenia^17–19^. Additionally, germline CNVs and their embedded miRNAs (CNV-miRNAs) were shown to be associated with autism^20^, roles in brain aging and neurodegeneration^21^ and congenital heart disease^22^. Prior studies have predicted that the target genes conferring the phenotypes are likely regulated by CNV-miRNAs^19^. However, there is no direct experimental evidence to support this premise.

**Figure 1:**
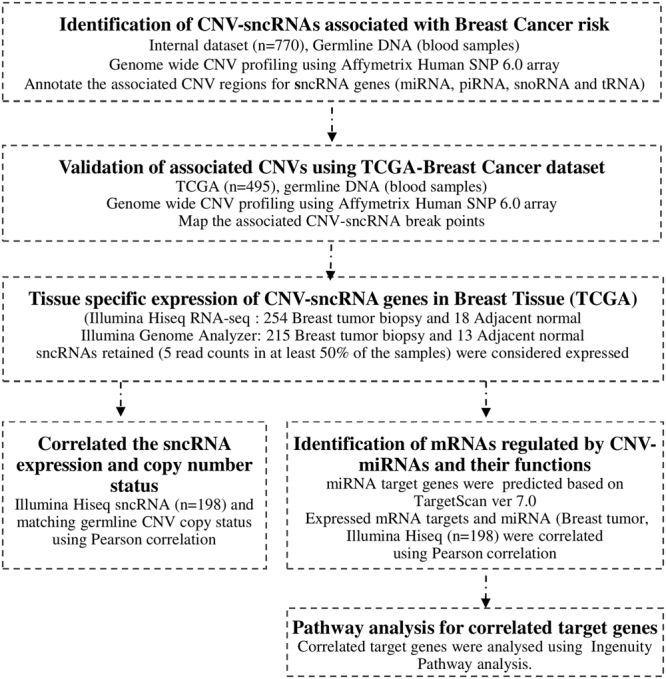
Schematic of the study design adopted. The flowchart depicts the overall study design, summary of the datasets, and experimental platforms used at each stage of the analysis. Detailed protocols and data analysis methods are discussed in the methods section.

We hypothesized that germline CNVs are associated with the phenotype of breast cancer, and that CNV-sncRNAs are indeed expressed in breast tissues, show gene dosage effects and mediate the regulation of downstream target genes. We show evidence in support of this hypothesis and offer insights on the role of disease associated CNVs. Firstly, we identified germline breast cancer associated CNVs using a genome wide association study (GWAS) design (Fig. 1) and identified embedded sncRNA gene regions. Secondly, we showed that sncRNAs originating in CNVs are indeed expressed in breast tissues and show correlation with germline copy status. Thirdly, we identified the target mRNAs regulated by CNV-miRNAs. We therefore infer that cancer associated CNVs harboring sncRNAs contribute to the pathogenesis of breast cancer.

## Results

### Identification of germline CNVs encompassing sncRNA genes and their association with breast cancer risk

**Figure 2:**
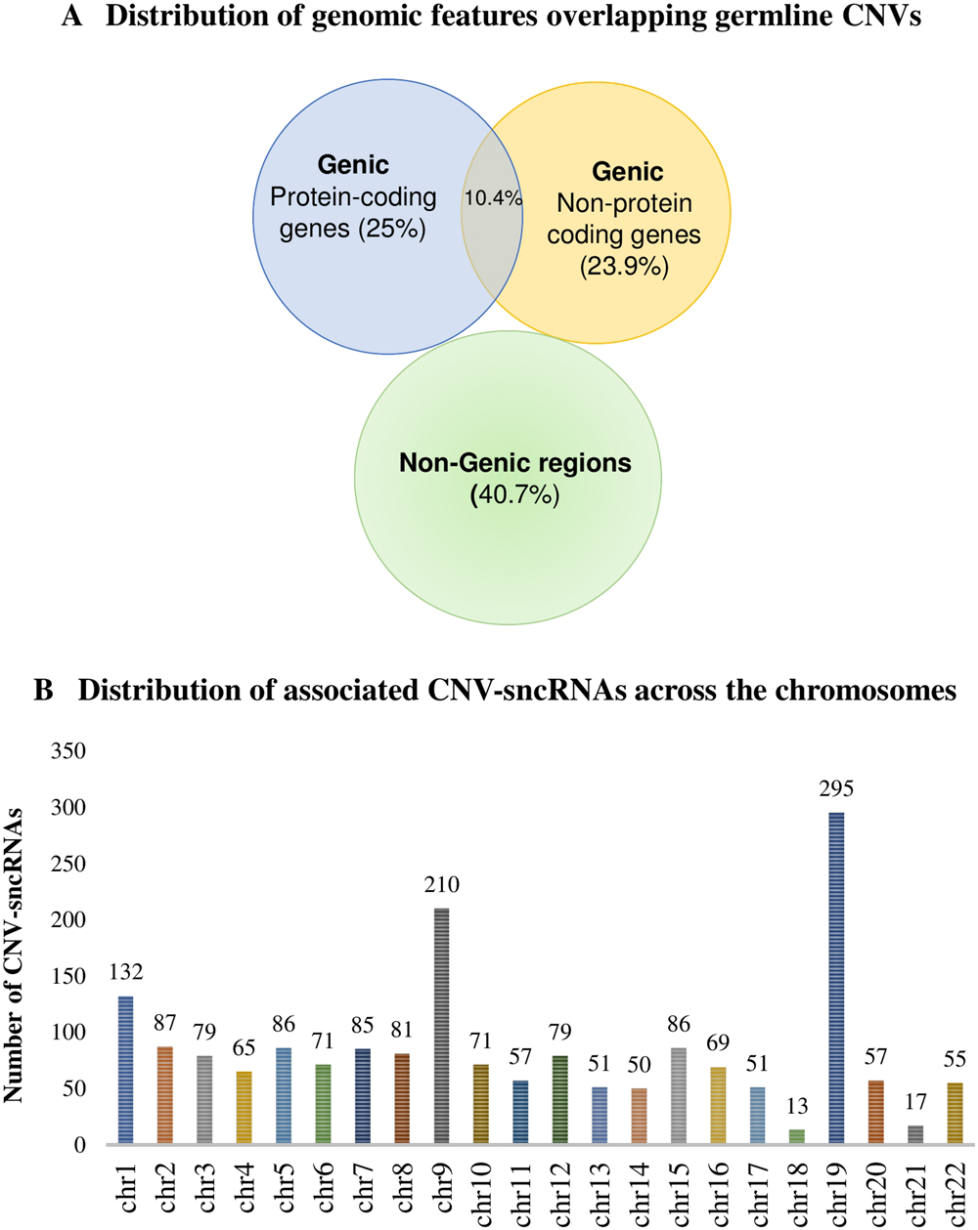
Genome wide distribution of germline CNVs. (**A**) Distribution of genomic features overlapping germline CNVs. Figure shows a Venn diagram of the genome wide distribution of germline CNVs associated (q < 0.05) with breast cancer. Represented genic regions were: protein coding (25%) and non-protein coding genes including pseudogenes and small and long non-coding RNAs (23.9%). An overlap of these regions (10.4%) capture non-coding RNAs originating from the intronic regions of the coding genes. 40.7% of CNVs do not show embedded genes (genome build hg19), hence labelled as non-genic regions. (**B**) Distribution of associated CNV-sncRNAs across the chromosomes. Figure illustrates the distribution of breast cancer associated CNVs (q < 0.05) harboring small non-coding RNA genes (miRNA, piRNA, tRNA and snoRNAs) for all chromosomes.

We conducted a GWAS (discovery dataset) using 366 cases/320 controls and germline CNVs as polymorphic markers. We identified 7496 CNVs that were associated with breast cancer risk (q-value < 0.05)^7^. Of these, 59.3% of the CNVs mapped to genic regions including protein coding genes, non-coding RNA genes and pseudogenes and the remaining 40.7% mapped to the non-genic regions. Among, the CNVs mapping to the genic regions, 25.0% (n = 1876) mapped to protein coding genes and another 23.9% CNVs (n = 1789) mapped to non-coding RNA genes, including genes for long non-coding RNAs, sncRNAs and to pseudogenes. We observed that 10.4% of the breast cancer associated CNVs (n = 776) mapped to both protein coding and non-coding genes because introns of the protein coding genes also serve as a source of non-coding RNAs **(**Fig. 2A). We have earlier described CNVs with embedded protein coding genes and their relevance to breast cancer^7^. Of the total 2565 CNVs (1789 non-coding RNA genes plus 776 non-coding RNA genes originating from protein coding introns), we considered 1812 CNVs harboring four classes of sncRNA genes (miRNAs, piRNAs, snoRNAs and tRNAs) for further analysis as these are known to play a role in post-transcriptional gene regulatory mechanisms.

The distribution of sncRNA genes within the 1812 breast cancer associated CNVs included miRNA (n = 38) and tRNA genes (n = 15), embedded within 26 and 10 CNVs, respectively. Each of the miRNA and tRNA genes that mapped within CNVs were non-redundant, in that none originated from multiple chromosomal locations. In contrast, piRNAs and snoRNAs showed redundancy, in that the same piRNA or snoRNA genes were found within multiple CNV loci across chromosomes. For instance, 9865 redundant piRNA genes were mapped to 1760 CNVs regions, of which 1292 piRNAs were unique. Seventy-one (or 66 non-redundant) snoRNAs were mapped to 52 CNV regions. **(**Supplementary Table S1). Individual frequencies of CNVs in cases and controls as well as the copy gain or copy loss frequencies are also summarized to facilitate comparisons. The average size of the associated CNVs was about 25 kb (range 50 bp to 9 Mbp). The number of sncRNA genes present within a CNV varied from 2 and 240, depending on the size of the CNV. About 36 CNVs harbored more than one class of sncRNAs, and piRNAs genes were predominant (Supplementary Table S1). Chromosomes 19, 9 and 1 showed the highest number of breast cancer associated CNVs, (295, 210 and 132, respectively), harboring sncRNAs (Fig. 2B), relative to other chromosomes. In summary, we have not only identified CNVs associated with breast cancer risk across the genome, but also the embedded CNV-sncRNAs.

We identified CNVs that overlapped with SNORD-115 and SNORD-116 clusters (chr15: 25296245-25326762) and were found to be associated with breast cancer (Supplementary Table S1). Deletion of these clusters were initially described in patients with Prader-Willi Syndrome (PWS)^23^. In our study, the SNORD locus showed both copy-gain (5–14%) and copy-loss (3–8%) in the cases but not in controls.

### Validation of CNV breakpoints in TCGA dataset

GWAS (n = 686, discovery stage) allowed us to identify CNVs (with embedded sncRNAs) that are associated with breast cancer risk. We used the TCGA cohort as a validation dataset to address the following: Firstly, to validate the CNVs from the discovery stage GWAS and to assess the replicability of copy number estimates between the datasets called by the same algorithm. Secondly, to examine breast tissue specific expression of sncRNAs embedded within CNVs. Thirdly, to identify regulatory potential of miRNAs (subset of all sncRNAs identified) using mRNA expression dataset from the same breast tumors from which sncRNAs were profiled.

We successfully mapped the 1812 CNVs (with embedded sncRNAs) from the discovery dataset to the TCGA dataset, thus validating the copy number estimates called by the algorithm (Supplementary Table S2). For comparisons of CNV break points in the discovery and TCGA data sets, we defined 100% overlap as those CNVs that had break points exactly matching or embedded within CNVs identified from either of the datasets. CNVs may have an influence on the level of expression of sncRNAs, and regulation of their downstream target mRNAs by diverse mechanisms. There is evidence to suggest that CNVs overlapping miRNA genes are more likely to exhibit phenotypic effects^24^, and we now extend this premise for other sncRNAs. Subsequent data analysis was based on TCGA cohorts for breast tissue expression analysis of sncRNAs and mRNAs from the matched samples.

### Breast tissue specific expression of the CNV-sncRNAs in TCGA dataset

**Figure 3:**
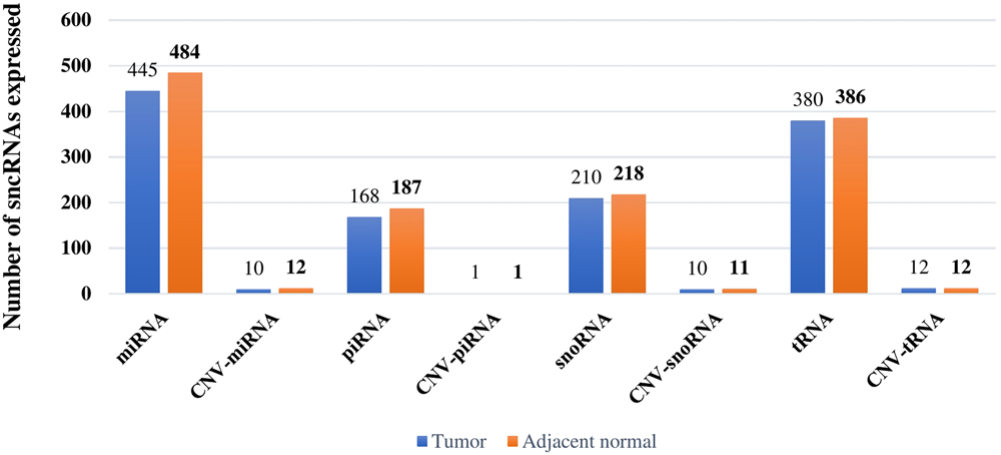
Expression profiles of small non-coding RNAs in breast tumor and adjacent normal tissues (HiSeq). Figure illustrates the expression profiles from the four classes of sncRNAs between tumor and adjacent normal tissues. Individual bar graphs capture the expressed total sncRNAs and CNV-sncRNAs. Data presented is from TCGA Illumina Hiseq (n = 254 cases and 18 adjacent normal).

Detailed analysis of sncRNAs identified in breast tumors and adjacent normal tissues using HiSeq (n = 254) and Genome Analyzer, (GA) (n = 215) platforms are summarized in Supplementary Table S3. Breast tissue specific expression of sncRNAs (miRNAs, piRNAs, snoRNAs and tRNAs) were analyzed. We compared the total number of sncRNAs expressed with the total number of sncRNAs originating from within the CNV regions. The total number of sncRNAs expressed were comparable between normal and tumor tissues. Similarly, we have also compared the total number of CNV-sncRNAs showing expression in normal and tumor tissues. (Figure 3). Overall, we have identified 38 CNV-sncRNAs (14 miRNAs, 1 piRNA, 11 snoRNAs and 12 tRNAs) expressed in both breast tumors and adjacent normal tissues. While CNV embedded snoRNAs, tRNAs and piRNAs were expressed similarly in both tumor or adjacent normal tissues, a subset of miRNAs detected were present either in tumor or normal tissues. Five of the miRNAs (hsa-miR-154-3p, hsa-miR-4999-5p, hsa-miR-382-3p, hsa-miR-487a-5p, hsa-miR-539-5p) were expressed only in adjacent normal tissues, at the cut-off criteria of 5 read counts in 50% of the samples. Using a similar cut-off criterion, one miRNA (hsa-miR-4746-5p) was expressed only in tumor tissues (Supplementary Table S4). A higher number of piRNA genes mapped to the breast cancer associated CNVs. However, CNV-piRNA, hsa-piR-20636 was the only one expressed in breast tumor tissue. In case of the snoRNA, we noted the C/D box SNORD 116 from the PWS loci showed expression in both breast tumors and adjacent normal tissues.

Breast cancer associated CNV regions showing overlap between discovery and validation datasets, and harboring the embedded sncRNAs (n = 38) are summarized (Table 1). It is interesting to note that 27% of CNVs (showing expression of embedded sncRNAs) were also reported as copy variable regions in the 1000 Genomes Phase 3 Project. A majority of the CNV frequencies were higher in cases relative to controls, thereby explaining the limited overlap with the 1000 Genomes data which is generated from the control populations.

**Table 1:** Germline CNVs in discovery cohort showing association with breast cancer risk and expression of embedded small RNAs in breast tumor tissues from TCGA.

Discovery Dataset							TCGA Dataset	
CNV region	Cytoband	length (bps)	p-value	q-value	CNV frequency gain/loss (%)		CNV region	Small RNAs expressed in breast tissues
					Cases	Controls		
*chr14:101513466–101514318	14q32.31	853	7.71E-05	9.21E-04	5/1	0/0	chr14:101513466–101517099	hsa-miR-539-5p (+), hsa-miR-889-3p (+)
*chr14:101515194–101519779	14q32.31	4586	4.84E-05	6.52E-04	5/1	0/0	chr14:101513466–101517099; chr14:101517099–101527707	hsa-miR-655-3p (+), hsa-miR-487a-5p
*chr14:101519779–101525402	14q32.31	5624	5.53E-05	7.27E-04	5/1	0/0	chr14:101517099–101527707	hsa-miR-134-3p (+), hsa-miR-134-5p (+), hsa-miR-323b-3p (+), hsa-miR-382-5p (+), hsa-miR-485-3p (+), hsa-miR-382-3p
*chr14:101525779-101527707	14q32.31	1929	8.94E-04	5.41E-03	4/1	0/0	chr14:101517099-101527707	hsa-miR-154-3p (+), hsa-miR-154-5p (+),
chr19:4437681–4494605	19p13.3	56925	3.09E-04	2.53E-03	3/2	0/0	chr19:4424993–4664433	hsa-miR-4746-5p (+)
chr1:149676729–149684202	1q21.2	7474	9.33E-06	1.77E-04	2/5	0/16	chr1:149676729–149684202	hsa-piR-20636
chr15:25296245–25297449	15q11.2	1205	4.32E-04	3.26E-03	5/1	0/0	chr15:25296245–25297449	snoRNA_SNORD116-1-201 (+)
chr15:25297449–25300158	15q11.2	2710	5.92E-07	1.92E-05	8/1	0/0	chr15:25298903–25300158	snoRNA_SNORD116-2-201 (+)
*chr15:25300158–25306451	15q11.2	6294	2.26E-07	8.49E-06	9/1	0/0	chr15:25300158–25304384; chr15:25305396–25308383	snoRNA_SNORD116-3-201 (+)
chr15:25307985–25310508	15q11.2	2524	6.12E-08	2.82E-06	9/1	0/0	chr15:25305396–25308383; chr15:25308383–25310928	snoRNA_SNORD116-6-201 (+)
chr15:25310508–25316405	15q11.2	5898	9.95E-08	4.25E-06	9/1	0/0	chr15:25310928–25318258	snoRNA_SNORD116-8-201 (+)
chr15:25316405–25318258	15q11.2	1854	2.62E-07	9.64E-06	8/1	0/0	chr15:25310928–25318258	snoRNA_SNORD116-9-201 (+)
chr15:25318258–25324279	15q11.2	6022	9.95E-08	4.25E-06	8/2	0/0	chr15:25318258–25325686	snoRNA_SNORD116-9-201 (+),
chr15:25324512–25325686	15q11.2	1175	2.87E-06	6.76E-05	6/2	0/0	chr15:25318258–25325686	snoRNA_SNORD116-14-201 (+)
chr15:25325686–25326762	15q11.2	1077	4.61E-06	9.87E-05	6/1	0/0	chr15:25325686–25326762	snoRNA_SNORD116-15-201 (+)
chr16:2011427–2016398	16p13.3	4972	6.98E-04	4.58E-03	3/2	0/1	chr16:2011427–2016398	snoRNA_SNORA10-201 (−), snoRNA_SNORA64-201 (−)
chr19:3975155–3984201	19p13.3	9047	3.09E-04	2.53E-03	3/2	0/0	chr19:3768181–4110048	snoRNA_SNORD37-201 (−)
chr1:148580449–148606453	1q21.2	26005	7.50E-09	4.65E-07	7/14	10/32	chr1:148580449–148632305	chr1.trna108-AsnGTT (−)
chr1:148705208-148768557	1q21.2	63350	7.26E-04	4.72E-03	4/11	4/22	chr1:148662374–148789654	chr1.trna107-AsnGTT (−)
chr1:149598086–149617469	1q21.2	19384	4.48E-10	4.08E-08	9/12	2/29	chr1:149598086–149631220	chr1.trna30-AsnGTT (+),
chr1:149661965-149670179	1q21.2	8215	3.70E-06	8.35E-05	4/8	1/19	chr1:149652461–149676729	chr1.trna94-GluTTC (−)
chr1:149670179–149676729	1q21.2	6551	3.60E-06	8.17E-05	2/6	0/17	chr1:149652461–149676729	chr1.trna92-PheGAA (−)
chr1:149676729–149684202	1q21.2	7474	9.33E-06	1.77E-04	2/5	0/16	chr1:149676729–149684202	chr1.trna90-ValCAC (−), chr1.trna91-GlyCCC (−)
chr6:26286287–26287456	6p22.2	1170	2.38E-04	2.13E-03	3/4	0/1	chr6:26274458–26287456	chr6.trna2-MetCAT (+)
*chr19:1381502–1407359	19p13.3	25858	1.23E-04	1.29E-03	4/2	0/0	chr19:1342160–1547869	chr19.trna1-AsnGTT (+), chr19.trna14-PheGAA (−)
*chr19:4658652–4771070	19p13.3	112419	3.09E-04	2.53E-03	3/2	0/0	chr19:4714925–4751218	chr19.trna13-ValCAC (−), chr19.trna2-GlyTCC (+)

This table represents the selected CNV regions associated with breast cancer that also included one of the four classes of sncRNAs. The statistics represented in this table is based on the discovery dataset (cases/control = 686) and includes the CNV region mapped in validation dataset (TCGA). These sncRNAs were expressed in the breast tissue (either breast tumor or adjacent normal tissues or both) in the TCGA dataset. The rows marked with *symbol indicates the CNVs that are also seen as copy number variable regions in 1000 genomes Phase 3 project.

### Correlation of expressed CNV-sncRNAs to copy status

CNVs are known to confer gene dosage affects among the protein coding genes^7,25^, and whether CNV-sncRNAs also show gene dosage effects was investigated. Correlation of the expression of the CNV-sncRNAs with corresponding copy status was addressed using Pearson Correlation analysis. Overall, 15 sncRNAs (one piRNA, eight tRNAs, six snoRNAs) showed correlation (Supplementary Table S5 and Supplementary Fig. 1); of these 13 correlated at p-value < 0.05 and two correlated at p-value < 0.1. One piRNA and five tRNAs showed positive correlation whereas three tRNAs and six snoRNAs showed negative correlations. The positively correlated sncRNA genes showed r = 14% to 21% and p-values 10^−2^ to 10^−3^. Negatively correlated snoRNAs showed r = −13% to −45% and p-values 10^−2^ to 10^−11^. Expression and regulation of sncRNAs are thus complex; while a positive correlation with copy status indicates potential gene dosage effects, a negative correlation may potentially indicate gene disruption or epigenetic regulation. This kind of negative correlations were also noted by others^26^ and there is no clear consensus mechanisms identified to explain these correlations. We observed that negatively correlated tRNAs originated from intergenic regions, whereas negatively correlated snoRNAs originated from intronic regions. We did not observe any significant correlations between copy status and miRNA expression. This could be due to the diverse mechanisms regulating miRNA expression. We could not distinguish if the CNV-miRNA itself is regulated by upstream elements within the CNV region or a combination of all the above.

### Gene targets for CNV-miRNAs and pathway analysis

We reasoned that a germline copy status for CNV-miRNA may show pronounced effects on downstream mRNA targets. To demonstrate such effects, we stratified breast cancer cases (mRNA expressions from n = 198 breast tumors from HiSeq Platform) based on germline status. Therefore, a correlation between miRNA and mRNA expressions may reveal higher number of targets that are regulated as a function of CNV copy status, as an indirect measure of miRNA copies. For instance, we examined CNV embedded hsa-miR-4746-5p in 198 breast cancer cases; 52 cases exhibited copy gains and 146 were diploid. Gene targets for the CNV-hsa-miR-4746-5p were predicted using TargetScan and these predicted targets were identified in the mRNA expression data sets (HiSeq platform). A correlation analysis revealed 25 common target genes for both diploid and copy gain cases; an additional 29 targets were identified for copy-gain cases (Supplementary Table S6). The miRNA-mRNA correlation (r) values were from −0.20 to −0.34; and from −0.27 to −0.42, for the diploid and copy gain cases respectively. The targets regulated by hsa-miR-4746-5p among the copy gain cases were enriched for key signaling molecules (growth hormone*, FLT3, NGF, PTEN*, G-protein coupled receptor) and glutamine biosynthesis pathways. The identified targets in our study have been well addressed in literature for their association with cancer^27–29^.

Except for the CNV region overlapping with hsa-miRNA-4746-5p, copy status for other nine CNV-miRNAs showed predominantly a diploid status, and therefore the correlation between miRNA and mRNA expressions were restricted to cases (n = 195) with diploid status (Supplementary Table S6). Ingenuity Pathway Analysis of the identified target genes regulated by hsa-miR-655, hsa-miR-134-3p, hsa-miR-4746 showed significant enrichment of several pathways (Supplementary Table S7). hsa-miR-655-3p and hsa-miR-134-3p had a common target gene, *DLD* (dihydrolipoamide dehydrogenase) which plays an important role in cellular biosynthesis and degradation of amino acid pathways. In addition, miRNA-134-3p targeted *CDK5* (Cyclin Dependent kinase 5)^30,31^, *POLE* (DNA polymerase epsilon, catalytic subunit)^32^ and *RAN* (member RAS oncogene family)^33^ with potential role in cell cycle.

## Discussion

GWAS approaches have identified several SNPs of low penetrance that contributed to the genetic risk of breast cancer^34–36^. However, the putative causal variants have not been identified for a majority of GWAS identified loci and thus limit our understanding of the role of these variants in disease etiology. CNVs are complex genomic variants which may show an overlap with protein coding and non-coding regions. Therefore, characterizing CNVs associated with breast cancer may offer potential mechanistic insights. CNVs can influence gene expression in several ways, including gene dosage effects and *cis*/*trans* regulation. In this study, we have addressed the role of germline CNVs with embedded sncRNAs in breast cancer. Although CNV embedded sncRNAs may play a role in disease pathogenesis, a direct demonstration of expression of sncRNA genes from CNV-sncRNAs was lacking^5^. This is the first study to identify associated CNVs containing four different classes of sncRNAs including miRNAs. We identified 1812 CNVs mapping to small RNA genes (38 miRNAs, 9865 piRNAs, 15 tRNAs and 71 snoRNAs) and significantly associated with breast cancer risk using a case-control approach. We gained insights into the associated CNV loci by quantifying the expression of the embedded sncRNA genes in both breast tumors and adjacent normal tissues.

sncRNAs play key roles in post-transcriptional gene regulation events, and variations in expression of sncRNAs may potentially affect their downstream targets. We identified a subset of CNV-sncRNAs that were expressed in both breast tumor and adjacent normal tissues. Since gene expressions are tissue specific, we expect only a small subset of sncRNAs to be expressed in breast tissues despite several sncRNA genes were annotated to the CNV regions. Recent studies on neurodevelopmental disorders have also identified CNVs were shown to be enriched with miRNA genes^17–21^. Several mechanisms have been proposed to explain the impact on the miRNAs based on the extent of CNV overlap with miRNA genes *e.g*., dosage effects attributed to loss of expression depending on the extent of overlap^24^. Other key findings of the study were as follows.

(i) Among the breast cancer associated CNVs (Table 1), four CNVs at 14q32.31 locus with embedded miRNA genes were confirmed as copy variable regions in the 1000 Genomes Phase 3 project. These CNV-miRNAs showed tissue specific expression in our study. Literature evidence suggests that regulated targets are influenced by levels of miRNA expression which in turn are regulated by feedback mechanisms^37^. Extending this premise, we reasoned that CNV-miRNA gene can potentially modulate expression levels and therefore affect downstream targets. However, we did not observe direct correlation of copy status and expression of the embedded-miRNAs. Instead, we observed that cases with germline copy gain regions with hsa-miR-4746-5p regulated more target genes than cases with diploid copy status for the same miRNA. Pathway analysis of the regulated genes indicated their involvement in cell cycle, receptor mediated signaling, proliferation and/or apoptosis.
(ii) piRNAs are known to play a role in maintaining genomic stability by repression of transposons through gene silencing mechanisms^38^ and are well studied in gonadal cells^39^. However, the role of piRNAs in somatic tissues and in cancer context are beginning to emerge. We showed piRNAs were differentially expressed between breast tumor and normal tissues and that piRNAs and their biogenesis pathway molecules (PIWI proteins) are prognostic^10^. miRNAs bind to the 3′-untranslated regions (UTR) of protein-coding genes and piRNAs also share similar mechanisms to mediate translational arrest or mRNA degradation^10^. In the Autism genetic database (AGD)^40^ which catalogs autism related CNV signatures, a higher proportion of CNVs harbored piRNA genes compared to other classes of small non-coding RNA genes. A similar trend was seen in this study wherein CNVs harbored several piRNAs compared to other sncRNAs, which cannot be fully attributed to multiple copies of piRNA genes. Instead, their tendency to be enriched in CNV regions may have evolutionary significance since earlier studies have noted that there are selective constraints on the origins of piRNA^41^ clusters in African populations. This is corroborated by the observed rates of insertion of transposable elements in African populations^17^. Although we mapped several piRNA genes to the breast cancer associated CNVs, only one (hsa-piR-20636) was expressed in both the breast tissues and showed trends of dosage effects. The functional significance of hsa-piR-20636 in the context of breast cancer warrants further studies.
(iii) We identified breast cancer associated CNVs (q-value < 10^−3^) overlapping with SNORD-115 and 116 clusters (15q11.2). Theses CNV were present only among breast cancer cases and showed a higher frequency of copy gain than copy loss. A previous study reported a CNV overlapping with the above loci at 15q11.2-13, spanning many protein and non-protein coding genes including the SNORD-115 and 116 clusters, which have been implicated in PWS^23^. In another study, wherein copy number gain in loci (chr15:24738239-24749581) upstream of the SNORD-116 cluster but in PWS loci was associated with obesity^42^. These findings suggest that copy gain or loss at these loci may confer diverse phenotypes including breast cancer. Genotyping platforms and CNV calling algorithms may contribute to the variation in the detected CNV breakpoints, therefore fine scale analysis is needed to confirm the exact breakpoints to delineate the mechanisms by which germline CNVs exerts pleotropic effects. We observed expression of eight snoRNAs from the SNORD116 cluster, and the expression of SNORD37, SNORA10 and SNORA 64 in both tumor and adjacent normal breast tissues. There are no known target RNAs regulated by SNORD116 in humans. However, SNORD 37 (target: 28S rRNA A3697) guides methylation, snoRNA 10 (target RNA: 18S rRNA U210 and 28S rRNA U4491) and SNORA 64 (target RNA: 28S rRNA U4975) directs pseudouridylation of the corresponding target rRNAs^43^. This supports the premise, that CNV embedded snoRNAs may play a role in regulation and maturation of the rRNA targets, although more direct experimental evidence is needed. Understanding the biological functions of these RNAs in the context of breast cancer susceptibility or tumorigenesis is needed.
(iv) tRNAs play a critical role in protein translation and previous studies have shown that expression of tRNAs and tRNA derived fragments were dysregulated in breast tumors^13^. Although the 1000 Genomes Phase 3 project has catalogued CNVs overlapping tRNA genes in the human genome, the role of germline CNVs with embedded tRNA genes was not studied in a disease context. Studies with model organisms demonstrated that copy number variation of tRNA genes alter the relative abundance of tRNAs, thereby altering codon usage^18,23,44,45^ and potentially stalling translation leading to formation of misfolded proteins^46,47^. The current study is the first to report the association of CNV-tRNAs with breast cancer and demonstrated their expression in breast tissues. Even though we correlated tRNA expression in breast tissues with germline copy status, our study limitation is in the direct extrapolation of findings to the tRNA abundance and their effects on translational mechanisms. Further, the weak but significant correlations observed between CNV status and tRNA expression levels may reflect the tight regulation. The primary goal of the study is to document that the germline CNV embedded tRNA genes are indeed expressed in breast tissues and that the germline CNV signatures show relevance to the etiological basis judging from the expression in tissues. While the current study focused on sncRNA, long non-coding RNAs are also known to regulate genes at the post-transcriptional level and their effects warrant independent investigations.

## Conclusion

In summary, we identified and validated germline CNVs associated with breast cancer. The break points identified in the discovery cohort were independently confirmed using the TCGA dataset. We were able to use the TCGA datasets since our discovery data set and the TCGA datasets were profiled for CNVs with the Affymetrix Human SNP 6.0 array platform. We acknowledge the potential limitation in the absolute calls of copy status due to differences in the control populations used as a reference. However, the unique aspect of the study was the integrative analysis of CNV calls, sncRNA and mRNA expressions in matched TCGA subjects. We showed that germline CNVs can potentially influence tissue level gene expression through their embedded sncRNA genes. Our findings provide a compelling rationale that germline CNVs have functional consequences, possibly mediated through gene dosage mechanisms.

## Methods

### Study ethics approval

The study was approved by the local Health Research Ethics Board of Alberta (HREBA) - Cancer Committee. Written informed consents were obtained from all study participants. All experiments performed using specimens from study samples were carried out under approved guidelines and regulation.

#### Study subjects and whole genome platforms

A schematic of the overall study design is summarized (Fig. 1) and details of the protocols followed are summarized below:

A: Discovery dataset: The study included women from Alberta, Canada with confirmed diagnosis of invasive breast cancer (cases, n = 422)^7,48^. The cases were non-metastatic at the time of diagnosis. Biological specimens and clinical-pathological information were accessed from the Alberta Cancer Research Biobank, located at the Cross-Cancer Institute, Edmonton, Alberta, Canada^49^.The controls (n = 348) included in this study were age matched healthy women (no personal or family history of cancer at the time of recruitment). The controls were accessed from a prospective cohort study called the Tomorrow Project^50^ based in Alberta, Canada. Affymetrix Human SNP 6.0 array data and information about the study participants and the specimens can be found elsewhere^34,48^ and in the ensuing text.

B: Validation dataset (The Cancer Genome Atlas Project, TCGA): We have accessed the dataset from TCGA study with cases diagnosed with invasive breast cancer. This study meets the publication guidelines provided by TCGA (http://cancergenome.nih.gov/publications/publicationguidelines). We accessed level 1 and level 3 TCGA datasets for Whole Genome Copy number profiles, small RNA sequencing data and mRNA sequencing datasets, respectively. The datasets were available for 1088 Invasive breast cancer cases. We selected 516 cases based on the study inclusion criteria: (i) no history of other malignancy, (ii) no metastasis at the time of diagnosis and (iii) diagnosis of invasive ductal or lobular carcinoma.

#### Germline CNV dataset from TCGA: Affymetrix Human SNP array 6.0 platform

We utilized Affymetrix generated (.CEL files) data from germline DNA. Based on the SNP genotype calls for the 516 cases, we performed population stratification analysis using Principal Component Analysis (PCA) as described in the ensuing text. We identified 495 cases with Caucasian ancestry which were used for the down-stream analysis.

#### Breast tissue transcriptome data set from TCGA for small non-coding RNAs: Next Generation Sequencing platform

We accessed datasets for small RNA sequencing files (level 1 data;.bam files) matching to 495 cases of Caucasian ancestry. Of these, sequencing data were available for 469 breast tumor tissues. However, for a subset of cases data were available on both tumor and adjacent normal tissues specimens. Sequencing data from Illumina HiSeq and Genome Analyzer (GA) platforms from TCGA were accessed (254 breast tumor samples and 18 adjacent normal samples from HiSeq and 215 breast tumor samples and 13 adjacent normal samples from GA).

#### Breast tissue transcriptome data set from TCGA for mRNAs: Next Generation Sequencing platform

We accessed mRNA sequencing data from breast tumors generated on Illumina HiSeq platform. Level 3 data (Reads Per Kilobase Million, RPKM normalized) was used for all analysis. mRNA sequencing data was available for 198 cases and these were matched with the data available for sncRNAs on the same HiSeq platform. This enabled the identification of post-transcriptionally regulated target mRNAs by CNV-miRNAs.

#### DNA extraction

DNA was extracted from peripheral blood samples of cases and controls (discovery dataset, n = 770). DNA isolation was carried out by using commercially available QiagenTM (Mississauga, Ontario, Canada) DNA isolation kits, as described earlier^34,48^.

#### Genotyping and Quality control

DNAs extracted from study samples was genotyped using Affymetrix Human SNP array 6.0 following manufacturer’s protocol and are described elsewhere^34^. Affymetrix SNP array 6.0 has an independent set of probes for SNPs and CNVs. Genotyping quality control was assessed using Birdseed V2 algorithm in Affymetrix genotyping console. Sample Contrast Quality Control (CQC) ≥ 1.7 indicates acceptable genotyping quality. All study samples (both discovery and validation data) had a CQC values >2.

#### Population stratification

Principle component analysis was performed using EIGENSTRAT algorithm implemented in Golden Helix SNP and Variation suite v8.5.0. Genotype data from 270 HapMap samples were used as reference to infer genetic ancestry of the study samples. Variance was accounted for by the top two principal components and a threshold of three standard deviations was set to determine the outliers.

Of the 770 samples in the discovery dataset, 686 samples co-clustered with the European ancestry samples from the HapMap data, and 84 samples were identified as outliers. Of the 516 TCGA samples, 495 samples were identified as belonging to the European ancestry and 21 samples were removed as outliers. Identity by descent (IBD) analysis did not reveal any cryptic relatedness among the study subjects as judged from the pair-wise correlation cut off <0.25 in both datasets.

#### Copy number estimation and association analysis

Copy Number Analysis was performed using Partek® Genomics Suite™ 6.6 (PGS) and the default parameters as described below. Affymetrix. CEL files served as the source files. The CNV analysis was performed for 686 samples (320 controls and 366 cases) and all sample normalization was used to create a reference baseline to infer the relative copy number estimate. Genomic segmentation algorithm implemented in the software was used to call the genomic segments based on the following default criteria: genomic markers >10; segmentation p-value threshold = 0.001; Signal/Noise (S/N) ratio = 0.3. The copy number status for each inferred segment was assigned based on the normalized intensity as diploid copy number = 1.7-2.3, copy gain >2.3 and copy loss <1.7. CNV association analysis was performed using 2 × 3 Chi-square association test estimates the difference in frequency of a CNV (gain/loss/diploid) between the cases and controls. Data was corrected for multiple hypothesis testing using Benjamin-Hochberg false discovery rate method and CNVs with q-value < 0.05 were considered significant.

CNV estimation for the 495-breast cancer TCGA samples (validation set) was performed similar to the discovery dataset, except for the normalization. We used HapMap 270 samples as a reference for a diploid status (controls) to infer copy status in TCGA samples (cases). Associated CNV regions and break-points from the discovery data set were mapped to the CNV profiles and break-points in TCGA samples.

#### Gene annotation for the CNV regions

Breast cancer associated CNV regions were annotated for sncRNAs from the following sources: mature miRNAs using miRBase ver20^51^, snoRNAs using Ensembl^52^, piRNAs using piRNAdb^53^ and tRNAs^54^ using UCSC genome browser. Protein coding and lncRNA genes were annotated using UCSC.

#### Expression analysis of sncRNAs

Partek® Genomics Suite was used for the analysis of sncRNAs and.bam files as a source of sequence data. TCGA samples (both breast tumor and adjacent normal tissues) sequenced using Illumina HiSeq platform and Genome Analyzer were analyzed separately using PGS. sncRNA annotation was based on the database sources described above. For sncRNA expression analysis, a cut-off at least 5 read counts in 50% of the samples was considered for further analysis. We restricted integrative analysis of CNV status, sncRNAs and mRNAs to HiSeq data because read depths may vary between HiSeq and GA platforms.

#### Correlation of the breast tissue expression of sncRNAs with germline copy number estimates

It was important to ascertain if there was a correlation between CNV copy status and expression of CNV embedded genes (e.g., encoding sncRNAs) in breast tumor tissues to assess the role of the latter in disease risk. We used Pearson Correlation analysis (p-value < 0.1) to demonstrate the relationship between copy status and sncRNA expression. We used 198 samples with germline CNV data and compared with sncRNA expression in matched breast tumor tissues from the TCGA cohort. sncRNA read counts (5 counts in at least 50% of the samples as a cut-off) were RPKM normalized and log-transformed to compare with the germline copy status as a categorical variable. Copy number status for each inferred segment was assigned based on the normalized intensity as diploid copy number (i.e., 1.7–2.3), with copy gain >2.3 and copy loss <1.7, as described above. Even though sncRNAs may originate from multiple genomic locations, we considered only expression of RNAs present within the breast cancer associated CNV regions.

#### Target predictions for miRNAs embedded within CNVs, tissue level mRNA-miRNA expressions and correlations with copy status

Target mRNAs for the 10 miRNAs were predicted *in silico* using TargetScan version 7.1. We accessed level 3 data for mRNA (HiSeq) from the TCGA cohort which is RPKM normalized and log-transformed. All of the predicted targets were expressed in the HiSeq mRNA data (albeit at varying expression levels). We performed RPKM normalization and log transformation of the miRNA expression data from HiSeq. The samples (n = 198) were initially classified into two groups based on their copy number status; Diploid and copy gains. Correlated mRNA-miRNAs were identified using Pearson Correlation coefficients and a negative correlation with r ≤ −0.2 and p-value < 0.05 was considered as indicative of regulated genes.

#### Ingenuity Pathway Analysis (IPA)

Data were analyzed using IPA (QIAGEN Inc., https://www.qiagenbioinformatics.com/products/ingenuitypathway-analysis) to identify potentially affected pathways. Coding genes targeted by miRNAs were used as an input to assess the pathways involved. Separate analysis was conducted for the genes identified in the stratified groups based on copy status. Enrichment p-value < 0.05 was considered significant.

## Electronic supplementary material

Supplementary Information

Supplementary Table S1

Supplementary Table S2
