# Supplementary material for: Breast cancer associated germline structural variants harboring small noncoding RNAs impact post-transcriptional gene regulation
Source: Sci Rep. 2018 May 14;8:7529. doi: 10.1038/s41598-018-25801-1 (PMC5951800; doi:10.1038/s41598-018-25801-1)
Supplement: Supplementary file 1 — Supplementary Information [file 41598_2018_25801_MOESM1_ESM.pdf]

Supplementary Information

Breast cancer associated germline structural variants harboring small noncoding RNAs impact post-transcriptional gene regulation

Mahalakshmi Kumaran[a], Preethi Krishnan[a], Carol E Cass[b], Roland Hubaux[e], Wan Lam[e], Yutaka Yasui[c] and Sambasivarao Damaraju[a,d,*]

Departments of [a]Laboratory Medicine & Pathology and [b]Oncology and the [c]School of Public Health, University of Alberta, Edmonton, Alberta, T6G 2R3, Canada; [d]Cross Cancer Institute, Alberta Health Services, Edmonton, T6G 1Z2, Alberta, Canada; [e]Department of Integrative Oncology, British Columbia Cancer Agency, Vancouver, BC, Canada

Supplementary Fig 1 Gene dosage analysis of CNV-sncRNAs

List of Tables S3-S7

- Table S3 NGS generated sequences and sncRNA annotations
- Table S4 List of 38 expressed sncRNAs (in TCGA dataset) embedded within the breast cancer associated CNVs
- Table S5 Gene Dosage analysis for CNV-sncRNAs
- Table S6 Gene targets for expressed CNV-miRNAs
- Table S7 Ingenuity Pathway Analysis for the target genes regulated by CNV-miRNAs

Supplementary Fig 1   Gene dosage analysis of CNV-sncRNAs

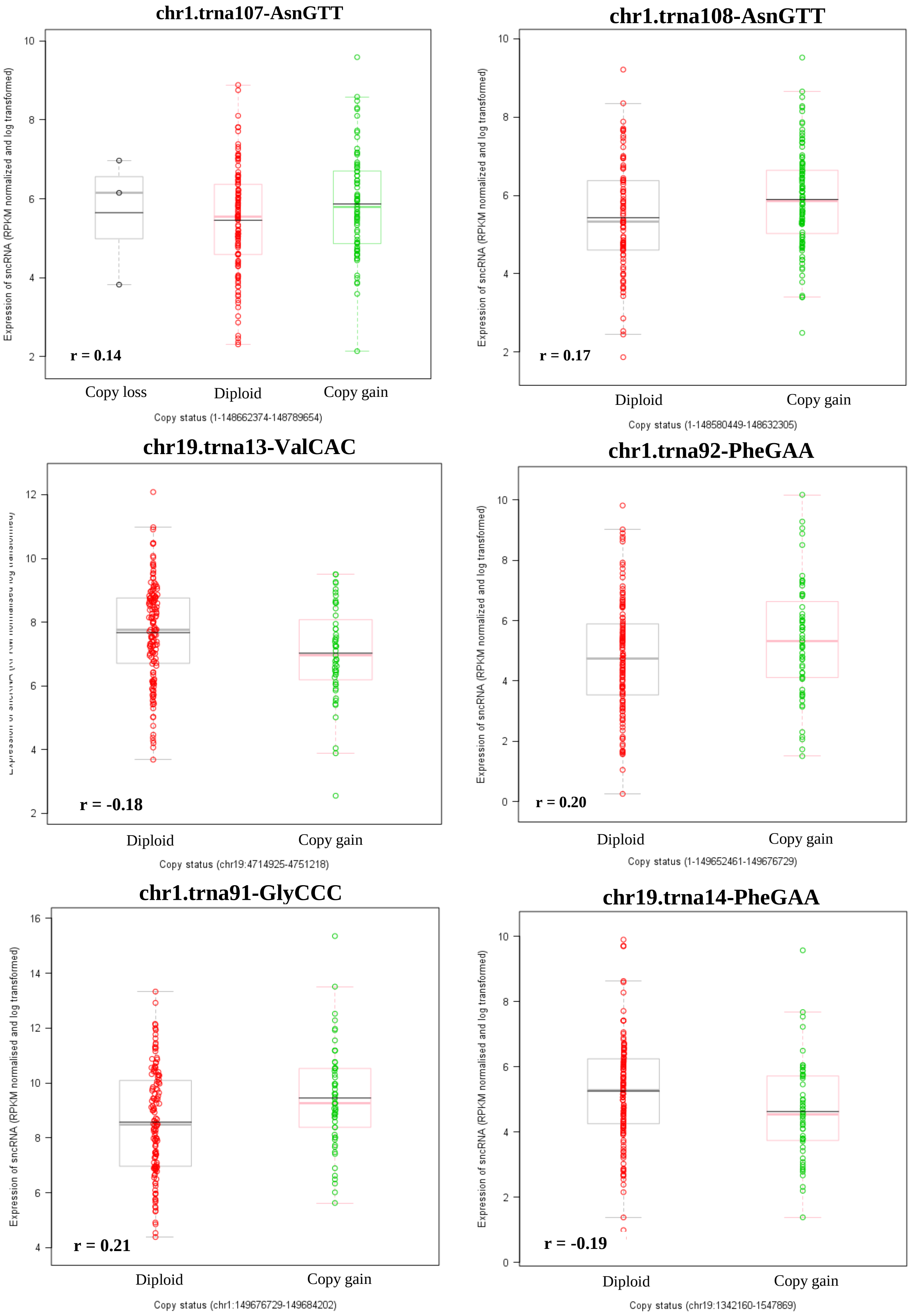

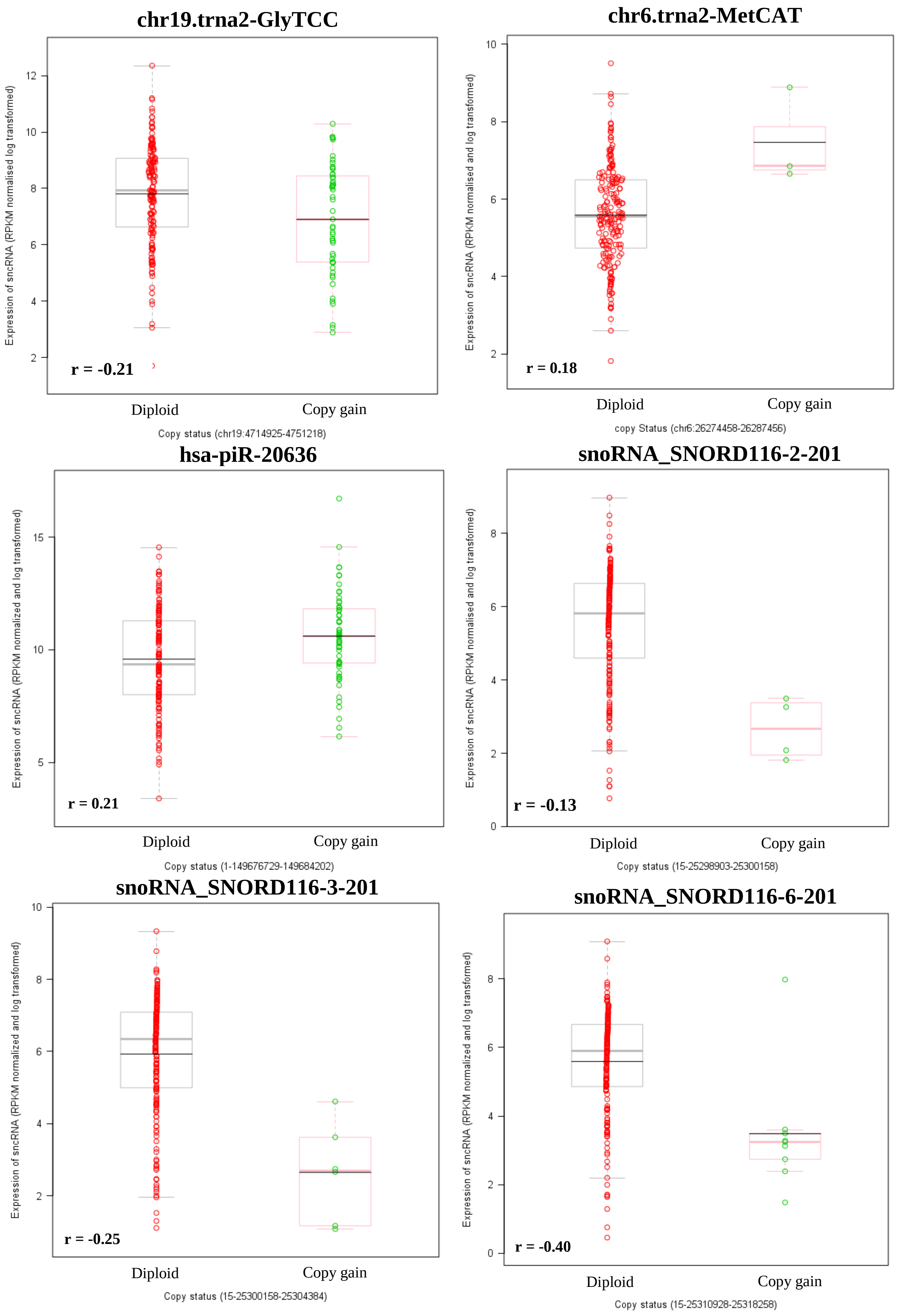
chr19.trna2-GlyTCC
Expression of sncRNA (RPKM normalised log transformed)
r = -0.21
Diploid
Copy gain
Copy status (chr19:4714925-4751218)
chr6.trna2-MetCAT
Expression of sncRNA (RPKM normalised and log transformed)
r = 0.18
Diploid
Copy gain
copy Status (chr6:26274458-26287456)
hsa-piR-20636
Expression of sncRNA (RPKM normalized and log transformed)
r = 0.21
Diploid
Copy gain
Copy status (1-149676729-149684202)
snoRNA_SNORD116-2-201
Expression of sncRNA (RPKM normalised and log transformed)
r = -0.13
Diploid
Copy gain
Copy status (15-25298903-25300158)
snoRNA_SNORD116-3-201
Expression of sncRNA (RPKM normalized and log transformed)
r = -0.25
Diploid
Copy gain
Copy status (15-25300158-25304384)
snoRNA_SNORD116-6-201
Expression of sncRNA (RPKM normalized and log transformed)
r = -0.40
Diploid
Copy gain
Copy status (15-25310928-25318258)

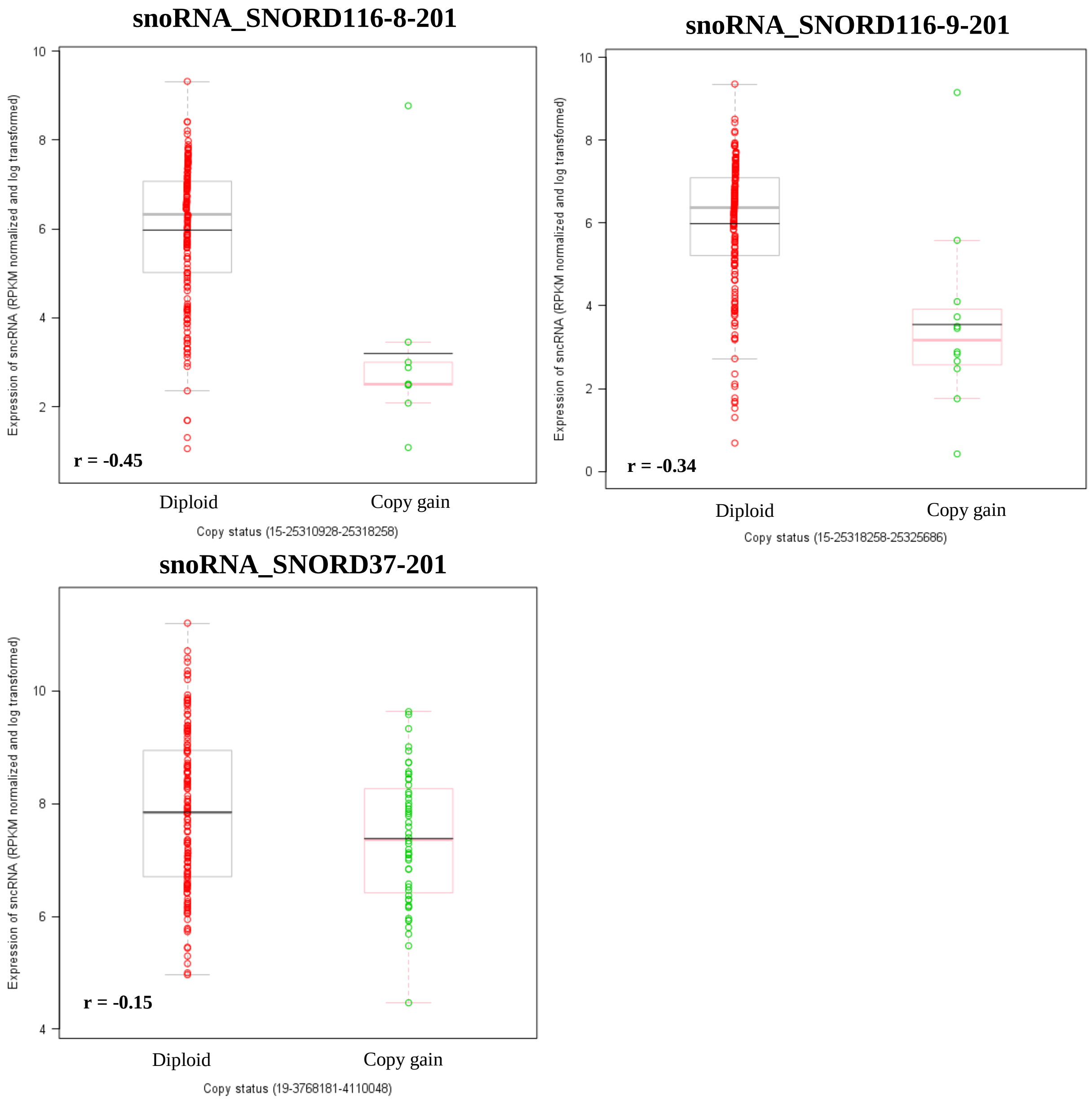

Gene Dosage for the embedded CNV-sncRNAs were estimated by correlating the germline copy status and sncRNA expression in breast tumor tissue data (HiSeq, n=198, RPKM normalized log transformed) using Pearson correlation. We observed significant positive and negative correlation among the correlated sncRNAs. Gray line in the plots represent the mean expression of sncRNA.

Table S3 NGS generated sequences and sncRNA annotations

Small RNA	Platform	Tissue type	No of samples	Total sncRNAs identified in tissues	No of sncRNAs retained after read count filtering	No of sncRNAs mapping to CNV regions
miRNA	HiSeq	Tumor	254	2235	445	10
miRNA	GA	Tumor	215	2068	360	7
miRNA	HiSeq	Adjacent normal	18	1616	484	12
miRNA	GA	Adjacent normal	13	1370	430	12
piRNA	HiSeq	Tumor	254	65074	168	1
piRNA	GA	Tumor	215	47695	147	1
piRNA	HiSeq	Adjacent normal	18	9325	187	1
piRNA	GA	Adjacent normal	13	4063	122	1
snoRNA	HiSeq	Tumor	254	1182	210	10
snoRNA	GA	Tumor	215	1001	201	10
snoRNA	HiSeq	Adjacent normal	18	665	218	11
snoRNA	GA	Adjacent normal	13	558	177	8
tRNA	HiSeq	Tumor	254	609	380	12
tRNA	GA	Tumor	215	597	364	8
tRNA	HiSeq	Adjacent normal	18	563	386	12
tRNA	GA	Adjacent normal	13	524	305	6

This table summarizes the result of sncRNA sequencing analysis, indicating the number of sncRNAs profiled in the tissue, number of sncRNA retained after read count filtering criteria (5 Read Counts (RC) in at least 50% of samples) and number of sncRNAs originating from the associated CNV regions. The results were summarized for tumor and adjacent normal tissues, as well as for the two sequencing platforms, Illumina HiSeq and Genome analyzer. There are no common samples between the two sequencing platforms.

Table S4 List of 38 expressed sncRNAs (in TCGA dataset) embedded within the breast cancer associated CNVs

chr	start	stop	strand	Small RNA	Tissue	Platform
14	101513666	101513688	+	hsa-miR-539-5p	Adjacent normal	HiSeq, GA
14	101514286	101514307	+	hsa-miR-889-3p	Tumor, Adjacent normal	HiSeq, GA
14	101515947	101515969	+	hsa-miR-655-3p	Tumor, Adjacent normal	HiSeq, GA
14	101518795	101518817	+	hsa-miR-487a-5p	Adjacent normal	HiSeq, GA
14	101520653	101520675	+	hsa-miR-382-5p	Tumor, Adjacent normal	HiSeq, GA
14	101520689	101520710	+	hsa-miR-382-3p	Adjacent normal	HiSeq, GA
14	101521031	101521053	+	hsa-miR-134-5p	Tumor, Adjacent normal	HiSeq, GA
14	101521069	101521092	+	hsa-miR-134-3p	Tumor, Adjacent normal	HiSeq, GA
14	101521801	101521823	+	hsa-miR-485-3p	Tumor, Adjacent normal	HiSeq, GA
14	101522606	101522628	+	hsa-miR-323b-3p	Tumor, Adjacent normal	HiSeq, GA
14	101526106	101526128	+	hsa-miR-154-5p	Tumor, Adjacent normal	HiSeq, GA
14	101526142	101526164	+	hsa-miR-154-3p	Adjacent normal	GA
19	4445984	4446007	+	hsa-miR-4746-5p	Tumor	HiSeq
19	8454224	8454245	-	hsa-miR-4999-5p	Adjacent normal	HiSeq
1	149680248	149680274	+	hsa-piR-20636	Tumor, Adjacent normal	HiSeq, GA
15	25296624	25296719	+	snoRNA_SNORD116-1-201	Tumor, Adjacent normal	HiSeq, GA
15	25299357	25299452	+	snoRNA_SNORD116-2-201	Tumor, Adjacent normal	HiSeq, GA
15	25302007	25302102	+	snoRNA_SNORD116-3-201	Tumor, Adjacent normal	HiSeq, GA
15	25310173	25310269	+	snoRNA_SNORD116-6-201	Tumor, Adjacent normal	HiSeq, GA
15	25315579	25315674	+	snoRNA_SNORD116-8-201	Tumor, Adjacent normal	HiSeq, GA
15	25318254	25318349	+	snoRNA_SNORD116-9-201	Tumor, Adjacent normal	HiSeq, GA
15	25325289	25325381	+	snoRNA_SNORD116-14-201	Tumor, Adjacent normal	HiSeq, GA
15	25326434	25326526	+	snoRNA_SNORD116-15-201	Tumor, Adjacent normal	HiSeq, GA
16	2012335	2012468	-	snoRNA_SNORA10-201	Tumor, Adjacent normal	HiSeq, GA
16	2012974	2013108	-	snoRNA_SNORA64-201	Tumor, Adjacent normal	HiSeq, GA
19	3982505	3982571	-	snoRNA_SNORD37-201	Tumor, Adjacent normal	HiSeq, GA
1	148598314	148598388	-	chr1.trna108-AsnGTT	Tumor, Adjacent normal	HiSeq, GA
1	148760356	148760430	-	chr1.trna107-AsnGTT	Tumor, Adjacent normal	HiSeq, GA
1	149608609	149608683	+	chr1.trna30-AsnGTT	Tumor, Adjacent normal	HiSeq
1	149664355	149664428	-	chr1.trna94-GluTTC	Tumor, Adjacent normal	HiSeq
1	149672905	149672977	-	chr1.trna92-PheGAA	Tumor, Adjacent normal	HiSeq
1	149680210	149680281	-	chr1.trna91-GlyCCC	Tumor, Adjacent normal	HiSeq
1	149684088	149684162	-	chr1.trna90-ValCAC	Tumor, Adjacent normal	HiSeq, GA
6	26286754	26286826	+	chr6.trna2-MetCAT	Tumor, Adjacent normal	HiSeq, GA
19	1383361	1383434	-	chr19.trna14-PheGAA	Tumor, Adjacent normal	HiSeq, GA

19	1383562	1383636	+	chr19.trna1-AsnGTT	Tumor, Adjacent normal	HiSeq
19	4724082	4724154	+	chr19.trna2-GlyTCC	Tumor, Adjacent normal	HiSeq, GA
19	4724647	4724720	-	chr19.trna13-ValCAC	Tumor, Adjacent normal	HiSeq, GA

This table lists the different classes of CNV-sncRNAs expressed in breast tumor and adjacent normal tissues, which were profiled using Illumina HiSeq and Genome analyzer platforms.

Table S5 Gene Dosage analysis for CNV-sncRNAs

CNV region	Expressed CNV-sncRNAs	Pearson Correlation Coefficient (r)	p-value (correlation)	No of samples
1-148662374-148789654	chr1.trna107-AsnGTT	0.14	5.44E-02	198
1-148580449-148632305	chr1.trna108-AsnGTT	0.17	1.40E-02	198
1-149676729-149684202	chr1.trna91-GlyCCC	0.21	3.52E-03	198
1-149652461-149676729	chr1.trna92-PheGAA	0.20	3.90E-03	198
19-4714925-4751218	chr19.trna13-ValCAC	-0.18	1.06E-02	198
19-1342160-1547869	chr19.trna14-PheGAA	-0.19	6.07E-03	198
19-4714925-4751218	chr19.trna2-GlyTCC	-0.21	2.94E-03	198
6-26274458-26287456	chr6.trna2-MetCAT	0.18	1.20E-02	198
1-149676729-149684202	hsa-piR-20636	0.21	2.64E-03	198
15-25298903-25300158	snoRNA_SNORD116-2-201	-0.13	7.29E-02	198
15-25318258-25325686	snoRNA_SNORD116-9-201	-0.34	1.03E-06	198
15-25300158-25304384	snoRNA_SNORD116-3-201	-0.25	3.66E-04	198
15-25308383-25310928	snoRNA_SNORD116-6-201	-0.40	5.05E-09	198
15-25310928-25318258	snoRNA_SNORD116-8-201	-0.45	2.46E-11	198
19-3768181-4110048	snoRNA_SNORD37-201	-0.15	3.16E-02	198

Gene Dosage for the embedded CNV-sncRNAs were estimated by correlating the germline copy status and sncRNA expression data (HiSeq, n=198, RPKM normalized log transformed) using Pearson correlation. We observed significant positive and negative correlation among the correlated sncRNAs.

Table S6 Gene targets for expressed CNV-miRNAs

miRNA	Copy status (no of samples)	No of Predicted and expressed targets	No of correlated targets	p-value	Pearson Correlation coefficient (r)	Correlated target genes considered for IPA analysis
hsa-miR-134-3p	Diploid (n=195)	4444	61	$<10^{-2}$	-0.20 to -0.27	*NAA40, TTF2, POLE,CDCA5,KDM2B,SETD8,ACP1,NCAPG2,TMED4,PGAM5,WDR77,DDX11,CDK5,GSG2,PTCD3,AGK,UBE2C,SRPK1,FARSB,SNRPD1,ELAVL1 DLD,RAN,USP13,TBRG4,C18orf25,PLCXD1,NUDT19,ZNF131,TROAP,VPS33A,DUS4L,TRIP13,RBBP4,ANKRD45,C11orf48,MOV10,ZNF695,FAM64A,MRS2,NUF2,DOCK3,PPIL1,MAP4K2,KNTC1,FBXO41,RSPO4,ABCF2,ZSCAN16,KIAA1549,NCAPH,FBRSL1,ZNF76,ATAD3B,ULK3,FANCA,RNF165,ATP5F1,PFDN6,PSMG1,FAF1*
hsa-miR-134-5p	Diploid (n=195)	176	3	$<10^{-2}$	-0.20 to -0.22	*DPH2, NIPA1, EXD1*
hsa-miR-154-3p	Diploid (n=195)	23	0			
hsa-miR-323b-3p	Diploid (n=195)	2638	0			
hsa-miR-382-3p	Diploid (n=195)	202	2	$<10^{-2}$	-0.20 to -0.25	*OCIAD2, HMGN3*
hsa-miR-485-3p	Diploid (n=195)	389	6	$<10^{-2}$	-0.20 to -0.22	*C18orf25, AGAP3, PEX5, FXR2, POM121C, POM121*
hsa-miR-539	Diploid (n=195)	3082	0			
hsa-miR-655	Diploid (n=195)	805	3	$<10^{-2}$	-0.20 to -0.22	*VKORC1L1, DLD, WHSC1*
hsa-miR-889	Diploid (n=195)	4339	0			
hsa-miR-4746	Diploid (n=146)	699	25	$<10^{-2}$	-0.20 to -0.34	*NRIP2, TXNDC15,NISCH,MXD4,CLEC14A,CD34,APBB2, ZNF446,EDNRB,RAX2,PCDH1,CDH5,ADAMTS13,AQP1,PALM,PDE11A,UNKL,F10,GIPR,PHF2,PDPK1,PHF1,LMX1B,NUDT16L1,AKAP12*

hsa-miR-4746	Copy gain (n=52)	699	54	< 10^{-2}	-0.27 to -0.42	*KLF10,NISCH,PCDH1,NRIP2,ZNF407,SLC35E2, CLEC14A,PTGER3,GIGYF1,ZNF423,UBR1,TBC1 D2B,CASZ1,IQSEC1,ADAMTS13,ADAMTSL1,CD H5,RAX2,CD34,LMX1B,ZBTB46,RPS6KA2,MXD4 ,ANKRD52,KBTBD11,CEP120,WDR81,SLC35E2,I GF1R,PDPK1,ERLIN2,EDNRB,LMTK2,MADD,P DE11A,MNT,ATOH8,CRX,CAMK2N1,CXorf23,CB X6,PHF2,KIAA1429,MOCS1,MGRN1,SERTAD1,S HOX,AQP1,ZHX3,ZBTB20,GLUL,PRDM2,KSR2,T MEM184A*

The table represents the targets identified to be putatively regulated by the CNV-miRNAs. The targets genes for each of the CNV-miRNAs were predicted using TargetScan. All the predicted targets were also expressed in the RNA-Seq data (RPKM normalized) from TCGA. The expressed target genes were correlated with CNV-miRNA. The miRNA-mRNA correlations with at least 20% (negative correlation) were considered significant. We have identified targets for the three CNV-miRNAs. For hsa-miR-4746, we performed the analysis for samples stratified based on copy number status (diploid vs copy gain) to understand the influence of CNV on the target regulation of hsa-miR-4746. We observed the copy gain group regulated more target genes in addition to the targets correlated in the diploid group.

Table S7 Ingenuity Pathway Analysis for the target genes regulated by CNV-miRNAs

miRNA name	Pathway	P-value	Target genes
hsa-miR-655 (Diploid)	Branched-chain α-keto acid Dehydrogenase Complex	5.62E-04	DLD
	2-ketoglutarate Dehydrogenase Complex	7.08E-04	DLD
	2-oxobutanoate Degradation I	7.08E-04	DLD
	Glycine Cleavage Complex	8.51E-04	DLD
	Acetyl-CoA Biosynthesis I (Pyruvate Dehydrogenase Complex)	9.77E-04	DLD
	Isoleucine Degradation I	2.00E-03	DLD
	Valine Degradation I	2.51E-03	DLD
	TCA Cycle II (Eukaryotic)	3.24E-03	DLD
	Super pathway of Methionine Degradation	4.47E-03	DLD
hsa-miR-134-3p (Diploid)	Cell Cycle Control of Chromosomal Replication	4.79E-03	CDK5, POLE
	Branched-chain α-keto acid Dehydrogenase Complex	1.10E-02	DLD
	2-ketoglutarate Dehydrogenase Complex	1.35E-02	DLD
	2-oxobutanoate Degradation I	1.35E-02	DLD
	Glycine Cleavage Complex	1.62E-02	DLD
	Acetyl-CoA Biosynthesis I (Pyruvate Dehydrogenase Complex)	1.91E-02	DLD
	BER pathway	3.24E-02	POLE
	NAD Phosphorylation and Dephosphorylation	3.47E-02	ACP1
	Isoleucine Degradation I	3.72E-02	DLD
	RAN Signaling	4.27E-02	RAN
	Valine Degradation I	4.79E-02	DLD
hsa-miR-4746 (Copy gain)	Growth Hormone Signaling	1.15E-03	IGF1R, PDPK1, RPS6KA2
	Glutamine Biosynthesis I	2.48E-03	GLUL
	FLT3 Signaling in Hematopoietic Progenitor Cells	1.89E-02	PDPK1, RPS6KA2
	IGF-1 Signaling	2.85E-02	IGF1R,PDPK1
	G-Protein Coupled Receptor Signaling	3.02E-02	PDE11A,PDPK1,PTGER3
	NGF Signaling	3.55E-02	PDPK1,RPS6KA2
	PTEN Signaling	3.55E-02	IGF1R,PDPK1

hsa-miR-4746 (Diploid)	Cardiac β-adrenergic Signaling	1.12E-02	AKAP12,PDE11A
	eNOS Signaling	1.41E-02	AQP1,PDPK1
	Extrinsic Prothrombin Activation Pathway	1.86E-02	F10
	Agranulocyte Adhesion and Diapedesis	2.04E-02	CDH5,CD34
	RAR Activation	2.09E-02	NRIP2,PDPK1
	cAMP-mediated signaling	2.82E-02	AKAP12,PDE11A
	Intrinsic Prothrombin Activation Pathway	3.39E-02	F10
	G-Protein Coupled Receptor Signaling	4.07E-02	PDPK1,PDE11A
	Coagulation System	4.07E-02	F10
	tRNA Splicing	4.47E-02	PDE11A

This table represents the findings from the IPA; represented are the pathways significantly enriched at p-value <0.05. For the hsa-miR-4746, we performed the analysis based on the targets identified in each of the copy number groups (diploid and copy gain). For the other two miRNAs, we used the identified targets genes based on cases with diploid copy status.
